# The state of genetic counseling supervision worldwide: Challenges, practices, and pathways for the future

**DOI:** 10.1002/jgc4.70169

**Published:** 2026-01-21

**Authors:** Lídia Guimarães, Bibiana Ribeiro, Margarida Rangel Henriques, Marina Serra Lemos, Milena Paneque

**Affiliations:** ^1^ i3S – Institute for Research and Innovation in Health University of Porto Porto Portugal; ^2^ IBMC – Institute of Molecular and Cellular Biology University of Porto Porto Portugal; ^3^ CGPP – Center for Predictive and Preventive Genetics University of Porto Porto Portugal; ^4^ ICBAS, School of Medicine and Biomedical Sciences University of Porto Porto Portugal; ^5^ AAJUDE – Associação de Apoio à Juventude Deficiente Porto Portugal; ^6^ FPCEUP – Faculty of Psychology and Educational Sciences University of Porto Porto Portugal; ^7^ CPUP – Center of Psychology University of Porto Porto Portugal

**Keywords:** continuous improvement, genetic counseling, patient‐centered care, professional development, professional support systems, reflective practice, safe practice, supervision

## Abstract

Genetic counseling supervision (GCS), a structured process in which a professional supports a supervisee through reflection, case discussion, and skills development, plays a crucial role in ensuring the quality and effectiveness of genetic counseling services worldwide. Despite its advantages, the implementation and consistency of GCS practices vary significantly across different regions, leading to disparities in the quality of genetic counseling services globally. The research was based on interviews with key professionals recognized as representatives of genetic counseling from diverse regions, including America, Europe, Asia, Africa, and Oceania. Data were analyzed using reflexive thematic analysis, identifying three overarching themes: (1) Historical and Structural Foundations of Access to GCS, including professional origins and institutional pathways, recognition of the profession, and policy, governance, and regulatory frameworks; (2) Meanings, Functions, and Perceived Value of GCS, encompassing its protective function against burnout, diversity of definitions and scope, and perceived contributions to practice quality; and (3) Forces Shaping the Implementation of GCS, covering genomics and increasing biomedical complexity, constraining pressures such as resources and workload, and enabling factors, such as networks, education, and digital solutions. These results indicate a promising shift toward broader recognition and integration of GCS, offering valuable insights for policymakers, genetic counselors and other healthcare professionals, and contributing to discussions on informed decision‐making and international collaboration.

**What is known about the topic?**

Genetic counseling supervision (GCS) is widely recognized as essential for professional development, quality assurance, and the delivery of patient‐centered care. It consists of a structured process in which a professional supports the supervisee through reflection, case discussion, and skills development. Current evidence highlights its role in strengthening clinical competence, fostering reflective practice, and guiding ethical decision‐making. Reported benefits extend to both practitioners, through enhanced confidence, reduced isolation, and continuous learning, and to patients, who receive safer and more consistent care.

**What does this paper add?**

This study provides global qualitative insights showing that formal recognition of the genetic counseling profession is a prerequisite for the development of GCS. The uneven recognition of the profession directly influences the establishment of GCS systems, while the absence of standardized guidelines and governance frameworks contributes to fragmented and inconsistent practices. GCS implementation is often challenged by resource constraints, administrative resistance, and clinical workload pressures, particularly in less resourced countries. International collaboration, professional associations, and the integration of GCS into training programs are key strategies to strengthen supervision worldwide. Finally, it reframes GCS as a long‐term investment in workforce resilience and patient care, an aspect of increasing relevance in the context of rapidly advancing genomic medicine.

## 1 INTRODUCTION

Genetic counseling supervision (GCS) is increasingly recognized as an important element in supporting the quality, consistency, and effectiveness of genetic counseling services across many countries worldwide (Costa et al., 2025; McEwen et al., 2025; Paneque et al., 2023). GCS is understood as a reflective process that fosters the continuous development of professional competencies. McCarthy Veach et al. (2003) describe it as a space to address emotional and ethical issues through the exploration of practices, feelings, and dilemmas. In line with this perspective, the National Society of Genetic Counselors (n.d.) defines GCS as a collaborative process that supports professional reflection while also emphasizing the emotional impact of counseling and the role of cultural factors in shaping counselor‐client interactions. Complementing these views, the American Board of Genetic Counseling (n.d.) characterizes GCS as a structured system of ongoing feedback that integrates both technical skills and emotional challenges, considering it essential for certification and the maintenance of professional competence.

Unlike informal case discussions or other types of supervision, GCS is typically formalized through a contract that specifies goals, frequency, mutual responsibilities, confidentiality, and professional boundaries. This contractual framework emphasizes that supervision is not a casual exchange but a deliberate commitment to sustained reflective practice and professional development (AGNC Supervision Working Group, 2007; McEwen et al., 2025).

To contextualize GCS conceptually, it is important to distinguish it from other supervisory models commonly used in healthcare and/or education. Clinical supervision, widely used in healthcare contexts, prioritizes the development of technical skills, performance evaluation, and case discussion in multidisciplinary teams (Bernard & Goodyear, 2008; Lindh et al., 2003; Scanlon & Weir, 1997). Educational or training supervision, typically introduced in academic or accreditation contexts, focuses on knowledge acquisition and the formative development of trainees (Ingvoldstad et al., 2016). In contrast, GCS addresses the ongoing needs of practicing professionals, placing particular importance on the psychosocial and emotional dimensions that are crucial in genetic counseling practice (AGNC Supervision Working Group, 2007; Lee et al., 2009). Fostering reflexive practice, GCS enables practitioners to integrate complex clinical experiences, enhance professional identity, and cultivate personal and professional resilience throughout their careers (Callanan & Ritchie, 2016; McEwen et al., 2025).

Research has highlighted the value of GCS for both practitioner well‐being and service quality. In the United States of America, Allsbrook et al. (2016) demonstrated that supervision can mitigate burnout and compassion fatigue, promoting resilience and job satisfaction among genetic counselors. Recent European studies similarly stress the emotional labor inherent to genetic counseling and the need for structured supervisory models that sustain reflexive practice and professional identity (Guimarães et al., 2024; Paneque et al., 2023). This growing body of evidence highlights not only the benefits of GCS for practitioners' well‐being but also its contribution to sustaining high‐quality and empathetic care delivery.

Multiple models of GCS have been described, reflecting diverse professional and personal needs. Individual supervision, for instance, enables in‐depth exploration of complex cases in a confidential one‐to‐one setting and might be important for new graduates (Kennedy, 2000; Lewis et al., 2017; Middleton et al., 2007), while group supervision fosters collaborative learning and peer feedback (Ferrer‐Duch et al., 2025). Peer supervision, though less formalized, offers mutual support and shared responsibility for reflexive practice (Hawkins & Shohet, 2006; Walker & Peterson, 2021). Some approaches also draw from systemic therapy, applying family systems techniques to supervision and exploring group dynamics and emotional processes, thereby mirroring the relational contexts of genetic counseling (Ferrer‐Duch, 2025; Hawkins & Shohet, 2006; McMahon, 2020; Middleton et al., 2007). Despite their differences, these models share the common goal of enhancing reflective capacity, resilience, and ethical awareness, ultimately improving counseling practice.

Despite this recognition, integration of GCS into professional frameworks remains inconsistent. In places such as the UK and Australasia, GCS is mandated and a requisite for certification or regulatory structures (AGNC Supervision Working Group, 2007; McEwen et al., 2025). However, in many European and non‐European contexts, access remains informal, optional, or ad hoc (Guimarães et al., 2024; Paneque et al., 2023). Such discrepancies block the recognition of GCS as a professional standard and limit its potential to systematically enhance quality of care, equity of access, and sustainability of the genetic counseling profession (Bernard & Goodyear, 2008).

This study aimed to investigate how GCS is implemented across different countries, to identify best practices, gaps, and strategies for implementation across diverse healthcare contexts. It is important to emphasize that this analysis represents only one component of a broader, worldwide investigation. Specifically, this manuscript focuses on aspects related to GCS, while a more detailed exploration of the status of the genetic counseling profession in the participating countries will be presented in a subsequent article.

## 2 METHODOLOGY

Semi‐structured interviews were selected for their flexibility and ability to capture in‐depth insights (Peters & Halcomb, 2015). This approach facilitates a comprehensive exploration of participants' experiences and perspectives, while also adapting to the diverse cultural and systemic contexts across countries. By combining guided questioning with open‐ended responses, the semi‐structured interviews allowed for the collection of detailed, nuanced data, essential for understanding the complex and varied nature of GCS practices worldwide (Peters & Halcomb, 2015). Additionally, this format supported a reflexive, interpretive approach, aligning with the principles of reflexive thematic analysis (RTA) (Braun & Clarke, 2019). In addition to the interviews, participants were invited to complete a brief demographic questionnaire. This questionnaire is provided in Appendix S1.

### 2.1 Participants

This study aimed to engage key representatives in genetic counseling worldwide to ensure a diverse and comprehensive perspective on GCS practices. Participants were recruited based on predefined inclusion criteria to ensure relevant expertise in the field.

To be eligible to participate in this study, individuals had to meet at least one of the following criteria:

- Be a representative member of a genetic counseling professional association.
- Be a member of an international board related to genetic counseling.
- Be a founder of a genetic counseling master's program.
- Be an advocate for the recognition and professionalization of genetic counseling in their country.
- Be a senior genetic counselor or geneticist with significant experience in the field.

### 2.2 Dataset generation

Study dissemination and participant recruitment occurred through two complementary approaches, aimed at maximizing outreach and ensuring a broad collection of interested individuals.

1. First, an email with an accompanying flyer outlining the study's purpose and key details was sent via the TAGC (Transnational Alliance for Genetic Counseling) mailing list to individuals identified as eligible. TAGC was selected for its global network of genetic counseling professionals and its strong role in education and professional development, making it an effective channel to reach engaged representatives. The email explicitly stated the eligibility criteria and invited those who met them to participate.
2. Second, at the World Congress of Genetic Counseling (WCGC) (2023) flyers were personally distributed to attendees, allowing for direct engagement and recruitment of potential participants during the event.

Individuals who demonstrated interest and met the eligibility criteria were then invited to complete a brief questionnaire designed to collect sociodemographic information relevant to the study (Appendix S1). The data collected included the participants' country of residence, their main professional activity, and years of experience in the field of genetics. Additionally, participants were asked to identify their profile according to the eligibility criteria. Participants were asked to suggest convenient dates for scheduling the interview. On the day before the scheduled interview, a link to the online interview and the interview guide were sent to the participants.

The interviews were conducted online via video conferencing, using the Microsoft Teams platform. Most interviews were held in English, while two were conducted in Portuguese and one in Spanish, based on the participants' language preference. It should be noted that not all interviews were conducted in the participants' native language, which may have introduced linguistic bias. None of the interviews were translated prior to analysis. All transcripts were analyzed in their original language to preserve linguistic and cultural nuances. Only the excerpts included in the manuscript from the Portuguese and Spanish interviews were translated into English for reporting purposes. These translations were carefully reviewed and verified by all three researchers to ensure accuracy and consistency with the original meaning.

At the beginning of each interview, the researchers introduced themselves, restated the study's objectives, and obtained permission to audio‐record the session, clarifying that the recording would be transcribed in real time. Informed consent was explicitly requested and verbally documented before proceeding. Participants were informed about the study's purpose, the voluntary nature of their involvement, and the confidentiality measures in place to protect their data. They were assured that their responses would remain anonymous and informed that they could withdraw from the study at any time. At least two of the three researchers involved in this research (MP; LG; BR) were present during the interviews: one served as the interviewer, while the other provided support, managing any technical issues or connection‐related concerns that might have arisen.

The study was approved by the i3S – Institute for Research and Innovation in Health, University of Porto, Portugal (16/CECRI/2021). Ethical approval ensured that the study adhered to necessary standards for confidentiality, informed consent, and voluntary participation.

### 2.3 Interview structure and content

The interview guide was divided into two main sections (see Table 1).

**TABLE 1:** Semi‐structured interview guide used to explore the implementation and characteristics of genetic counseling supervision worldwide.

Semi‐structured interview guide
Worldwide perspectives on genetic counseling supervision: An in‐depth exploration of implementation strategies, global practices and future directions across continents
**Part 1**
Brief exploration of the context of the genetic counseling profession in the country represented by the participant.
**Part 2**
Do you have GCS in the country you are representing? What are the format characteristics of the GCS: frequency, whether it is mandatory, whether it is performed in healthcare services or not and how it is funded? Do you know how the implementation process of the GCS occurred?

Abbreviation: GCS, genetic counseling supervision.

The first section briefly explored the context of the genetic counseling profession in the participant's country.

The second section focused specifically on GCS within each participant's country. Questions examined whether GCS was in place and explored its key characteristics, such as frequency, mandatory status, integration within healthcare services, funding mechanisms, and the process of its implementation.

### 2.4 Data analysis

The interviews, which lasted between 26 and 110 min with a median duration of 46 min, were audio‐recorded and transcribed using the Microsoft Teams transcription tool, allowing for simultaneous capture of participant responses. For the analysis of the interview transcripts, RTA was employed, both for its methodological rigor and for its suitability in exploring complex, multicontextual professional experiences in an interpretive and reflexive manner (Braun & Clarke, 2006, 2019, 2020, 2024). This approach enabled systematic identification, coding, and analysis of patterns of shared meaning within the data, providing insights into perceptions and practices of GCS worldwide.

The authors of this study are five women with diverse academic and professional backgrounds. Among the researchers, four have a background in psychology and one in genetics, with diverse levels of experience in the field. One is a recent graduate in genetic counseling. The second researcher is a genetics researcher with over 10 years of experience in the field. The third researcher is a senior genetic counselor, with extensive expertise in research and a Cuban immigrant residing in Portugal. The other two researchers are senior psychologists with extensive research experience and a strong presence in the academic field. The researchers recognize that their identities and distinct personal and professional trajectories shape their perspectives and interactions within the research process. This reflective stance acknowledges that researcher positionality is embedded in the analysis aligning with the reflexive nature of RTA, which emphasizes the researcher's active role in meaning‐making and interpretation (Braun & Clarke, 2006, 2019).

The analytic process followed Braun and Clarke's six‐step framework (2006). All transcripts were initially analyzed by two of the researchers (LG, BR) and initial codes capturing key features of the data relevant to the research questions were generated systematically. Subsequently, the initial conceptual diagrams created by these researchers were reviewed and discussed with a third researcher (MP). Codes were then examined for conceptual and contextual connections and grouped into preliminary themes, which were reviewed collaboratively to ensure coherence and distinctiveness. Themes were further refined and clearly labeled to reflect their core meanings, and a conceptual map was created to visually represent the relationships between themes and subthemes. The final analysis was written up with illustrative participant quotes, linking findings to the research questions and relevant literature.

Although some themes were less frequently mentioned or occurred only once, these were carefully considered for their potential relevance and contribution to a comprehensive understanding of the field. Consistent with RTA, themes were conceptualized as patterns of shared meaning. A checklist (Braun & Clarke, 2024), demonstrating how each item was addressed in the article, is provided as supplementary material.

## 3 ANALYSIS

### 3.1 Participants profile

Responses were collected from participants across 23 countries, representing five continents: Portugal, Greece, Romania, Austria, Spain, the United Kingdom, Iceland, Germany, Malta, France, Cyprus, Sweden, Brazil, the USA, Canada, India, South Korea, Taiwan, Israel, the Philippines, Australia, and several African countries, notably South Africa (see Table 2). These participants represented a broad geographical distribution spanning Europe, Asia, America, Africa, and Oceania, with a particularly strong representation from Europe.

**TABLE 2:** Participants profile.

Professional profile	Main professional activity	Number of participants with this profile
Founder of a genetic counseling master's program	Genetic counselor	7
Representative member of a genetic counselors' professional association	Genetic counselor	6
Senior professionals	Genetic counselor/medical geneticist/author, mentor, retired training program director	5
Advocate for the profession's recognition in the country	Genetic counselor/human genetics laboratory specialist and genetic counselor	5

**FIGURE 1:**
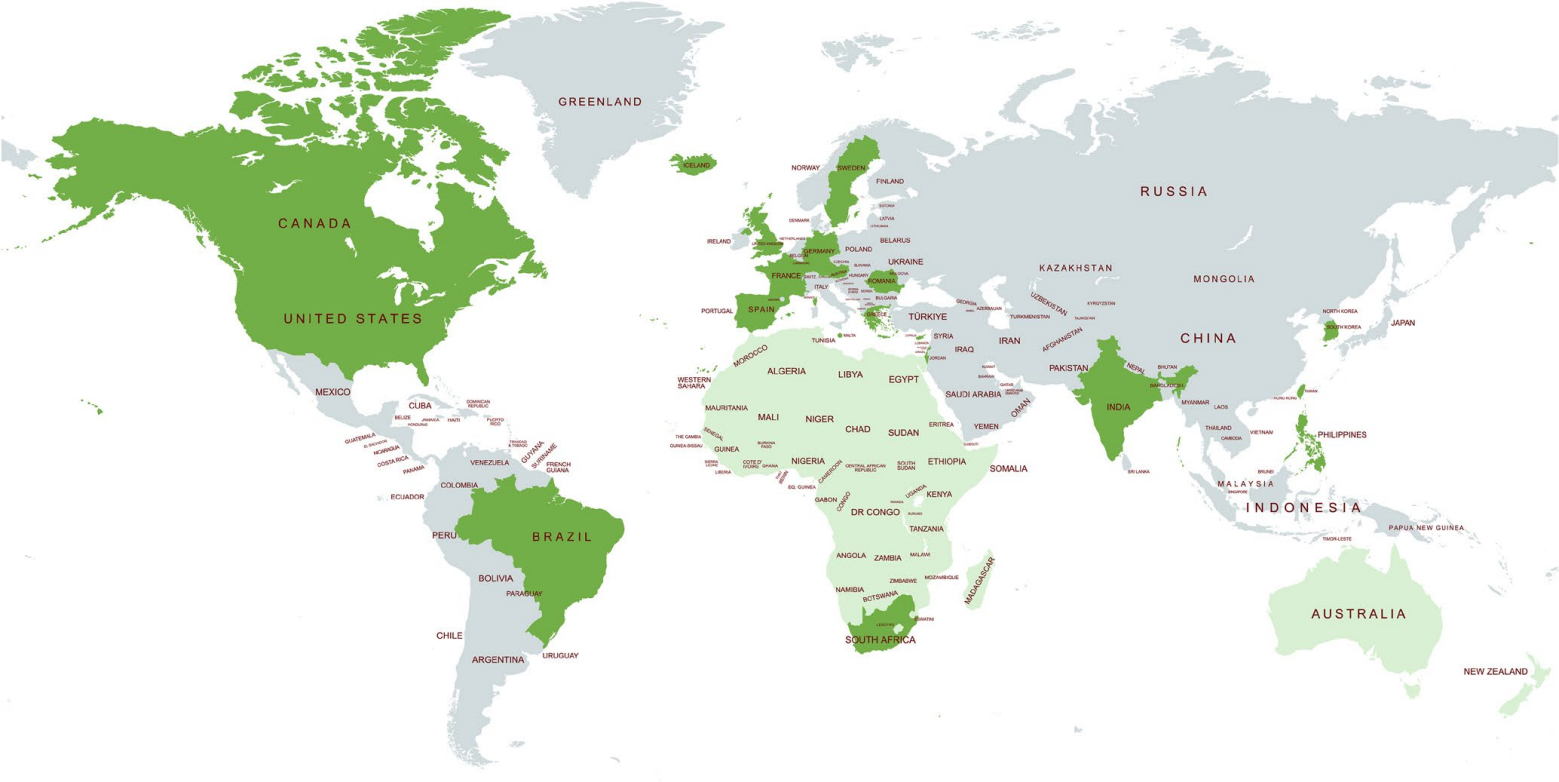
Geographical distribution of participants. Dark green: Country directly represented by a participant; light green: Regional input provided on behalf of multiple countries.

Participants had a wide range of professional experience. Eleven participants had over 21 years of experience, five had between 11 and 20 years, and seven had less than 10 years of experience in the field of genetics. Participants with over 21 years of experience frequently occupied leadership, advocacy, or educational roles, including founding genetic counseling master's programs and mentoring early‐career professionals. The map below (Figure 1) illustrates the geographical distribution of the consulted participants. Countries marked in dark green indicate those that were directly represented, while lighter green highlights regions or continents for which participants provided input on behalf of multiple countries.

In the brief contextual exploration conducted in Part 1, participants were asked to describe whether the genetic counseling profession was formally recognized in their country and, if so, for how long. Responses revealed considerable variation. Countries like Portugal, Austria, Spain, Germany, or Iceland described the profession as gaining visibility over the last decade, often supported by initial training initiatives and the first cohorts of professionals entering practice. By contrast, participants from Canada, the United States of America, the United Kingdom, and Australasia reported that the profession has been formally established or consolidated for more than 10 years, with accredited master's programs, professional associations, and systems of certification or regulation in place.

### 3.2 Global GCS landscape

**FIGURE 2:**
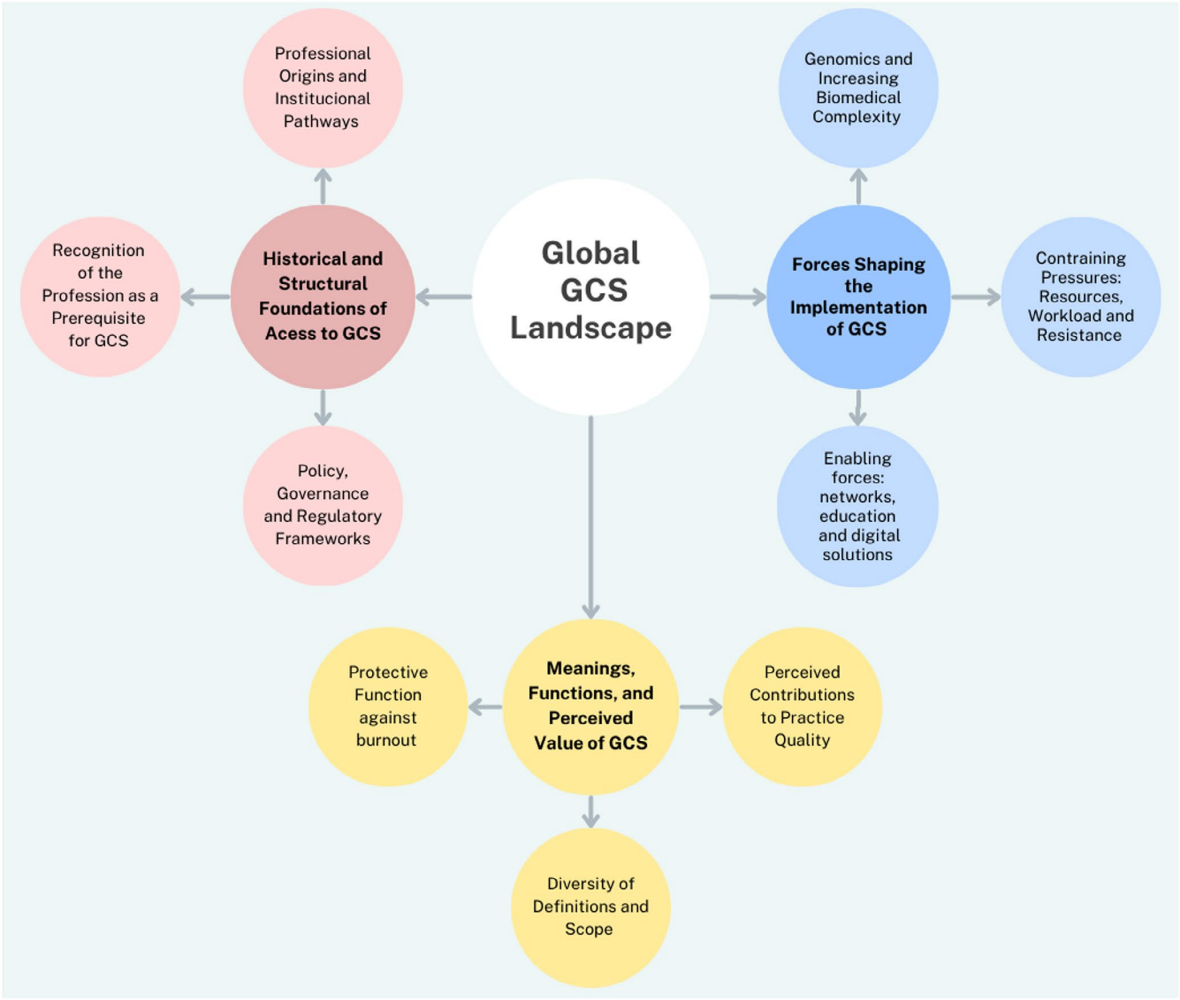
Conceptual map of the global genetic counseling supervision (GCS) landscape.

Following RTA, three overarching themes were constructed (Figure 2).

*Historical and structural foundations of access to GCS*—This theme captures how GCS practices are shaped by the historical trajectory of the genetic counseling profession. Key influences include the formal recognition of the profession, the evolving development of genetics, the legacies of early professional models, and relevant policy, governance, and regulatory frameworks.

*Meanings, functions, and perceived value of GCS*—The interviews revealed that GCS is understood and defined in diverse ways yet widely valued both for its contribution to practice quality and for its protective function against professional burnout.

*Forces shaping the implementation of GCS*—We interpreted participants' narratives as highlighting the tension between constraint pressures (such as workload, scarce resources, and managerial resistance) and enabling factors (including professional networks, collaboration, education, and technological innovations) that together shape the feasibility of GCS.

#### 3.2.1 Historical and structural foundations of access to GCS

Analysis of participants' accounts highlighted how access to GCS is shaped by interrelated historical and structural foundations, rather than by isolated professional practices. These foundational elements include the recognition of the genetic counseling profession, the institutional and regulatory framework in which it is embedded, and the professional legacies that continue to inform how GCS is valued and practiced.

##### Recognition of the profession as a prerequisite for GCS

Across settings, participants repeatedly stressed that the existence and development of GCS programs appeared to be intrinsically linked to the recognition of the genetic counseling profession. They described contexts in which official professional status allowed structured pathways for GCS to emerge, including institutional support mechanisms.

Every genetic center will have monthly supervision. Some have it twice a month. The professionals will have to have GCS to maintain the registration. In our center we have two hours every month for group supervision but, as I say, some centers will do it a little bit differently and some set centers will offer one‐to‐one supervision. And because it's necessary to maintain our registration, the department will budget it. (UK, GC)

In contrast, in contexts where the profession lacked recognition, participants reported that GCS was inconsistent or even absent, frequently attributing this absence to the lack of professional recognition.

We don't have GCS, but we have been thinking about it more because of what we have learned from other countries. (…) up until 2024 we were trying to train counselors and to get them to a professional level where we have certification processes and now, I think we are ready to think about genetic counseling supervision. (India, GC)

##### Historical legacies of the profession

Participants' accounts highlighted how the historical roots of genetic counseling seem to shape the perceived relevance of reflective practice and how it is practiced and valued in different contexts. Historical legacies are not approached here as descriptive professional histories, but as enduring professional orientations that continue to shape contemporary expectations of supervision. Participants' accounts illustrated how these historical roots continue to shape contemporary supervision practices, influencing both the content and purpose of GCS sessions and the expectations placed on supervisors and supervisees. The participants constructed that in settings where the profession initially developed in close connection with psychology, there is a stronger emphasis on reflective practice as a core element.

(…) A key moment, I think, was 1992. In the UK, genetic counseling has a longer history dating back to the 1970s and 1980s. With the incidence of spina bifida, a lot of community workers were involved in genetics. Then Lauren Carson‐Storr came over from the USA, having completed a master's programme at Berkeley, and she arrived in the 1980s to work in Manchester. (…) Her programme director was actually Seymour Kessler at the time, hence the interest in the psychosocial aspects of genetics, which, as you know, has been a key emphasis on the programme and which we try to maintain to this day. For us, the psychosocial part was incredibly important. (UK, GC)

In contrast, where the profession emerged primarily from a biomedical tradition, GCS tended to be treated as secondary rather than central to genetic counseling practice.

GCS isn't mandatory. I think this has to do with the origin of the profession in our country, which comes more from a biomedical background. In the UK, their profession seems to have emerged more from a psychological background, so they've adopted more of the professional practices and assessment approaches used in psychology and really emphasized and promoted the psychological aspects of the work (…). (Canada, GC)

##### Policy, governance and regulatory frameworks

Participants' accounts highlighted policy, governance, and regulatory frameworks as central structural foundations shaping access to GCS. In settings where formal governance structures for genetic counseling were established, GCS tended to be implemented consistently, following defined procedures that included regular scheduling and clearly delineated roles for supervisors and supervisees. Participants described how such frameworks not only legitimized GCS as part of professional practice but also facilitated institutional support and protected time for supervision.

To explain how GCS is in our region, I need to speak to you about our supervision policy. To practice as a genetic counselor and be on the register of genetic counselors in Australasia, you must have supervision: it's a mandatory part of being a registered genetic counselor. A fully qualified genetic counselor must have monthly supervision, one hour every month, which comes to a minimum of 10 hours per year. And then, for genetic counselors who've finished their master's degree but are still working towards their full registration, they also must have supervision, I think every two [weeks]. (Australasia, GC)

By contrast, participants working in settings without formalized governance described the presence of informal “routines” rather than structured or enforceable frameworks. In these contexts, GCS relied largely on collective goodwill and peer support, lacking the consistency and accountability associated with regulated systems:

We have our routines, but it's not like in the UK, where it's very well established that once a month you need to go. You know, we support each other with our own efforts, but the UK is very organized in that sense, as you had said: everything is written out, and it's written out because they have their policy makers following up on a lot of things, I mean. (Cyprus, GC)

#### 3.2.2 Meanings, functions, and perceived value of GCS

There remains considerable lack of clarity about what GCS entails, with many participants primarily associating it with clinical supervision or student supervision. These accounts highlight how understandings of GCS are shaped by professional experiences and local practices, rather than by shared or standardized definitions. Despite these differences, participants consistently described GCS as an essential resource supporting professional development and psychosocial well‐being, and as contributing to perceptions of high‐quality practice and continuity of services.

##### Diversity of definitions and scope

Participants reported misunderstandings about what GCS is, with many describing it mainly in terms of educational or clinical supervision, reflecting its integration into training rather than its application in ongoing professional practice. These accounts reveal not only conceptual ambiguity around GCS but also significant variability in how it is accessed and practiced across different settings.

Over a certain period, you have to this type of interaction with a genetic counselor or psychologist or someone. Obviously, this is integrated into our degrees. So, supervision for our students and our interns is a continuous consistent thing that forms part of their training. Now in an African Country the same thing happens. I'm the only genetic counselor. so obviously the only supervision that's happening… there's no regulation about whatever I'm doing or how I'm doing it. (Africa, GC)

We set up the conferences for all the genetic counselors to come together and share the process of genetic counseling. Because you run into this problem, I run into that problem, and different genetic counselors run into different problems. So, we have a fixed conference call, it's like quarterly. Our association set up a case conference so that every genetic counselor can provide their special case and share among the genetic counselors. (Taiwan, GC)

##### Perceived contributions to practice quality

Participants described GCS as supporting reflective practice, ongoing professional development, and maintenance of care quality. They highlighted that supervision allowed practitioners to review cases, discuss challenges, and refine both technical and interpersonal skills, supporting consistent and effective service delivery. For instance, a participant from Portugal emphasized the vital role of supervision in professional growth and service improvement, advocating for its formal integration into professional standards:

Therefore, supervision in counseling should be seen as a continuous source of professional growth and service improvement and should be mandatory. It also appears essential to link GCS to the formal recognition of the profession (…) and in the Code of Practice itself, when the profession gets recognize in our country, in the very requirements for practicing the profession. (Portugal, GC)

Similarly, experiences in the USA highlighted the personal and professional impact of effective supervision, where structured GCS sessions fostered a safe environment for discussion and reflection, enhancing practitioners' confidence and competence:

I was fortunate to be one of the first to assist GCS in the USA and it was an absolutely remarkable experience. The way in a short period of time that the supervisor was able to make us feel comfortable and talk about really important aspects of genetic counseling. There are issues of safety and confidentiality on one hand, but it was a remarkable experience. And then the next year they did it again because it was so successful, people were really interested in it. (USA, GC)

##### Protective function against burnout

Participants reported that supervision played a protective role against professional exhaustion and emotional strain, particularly by providing reflective and relational spaces within practice. They described that access to reflective spaces allowed practitioners to process challenging experiences, manage stress, work as a team, and maintain emotional well‐being. For example, a participant from the UK emphasized the role of supervision in promoting teamwork and preventing burnout, both for practicing professionals and for students:

I think GCS helps teamwork, and I think in the era of genomics it's going to be important that people don't burnout and that they work well as a team. I also think GCS helps with that (…) I worry about burnout for the professionals but also for the genetic counseling students, to be frank. But I would for anybody working in a public health service (…). (UK, GC)

Similarly, in some contexts, participants linked GCS to health and safety regulations, reinforcing its perceived role in safeguarding overall well‐being.

You can find kind of a mandate for GCS in health and safety policies in hospitals (…) because health and safety are about people's overall well‐being. (Africa, GC)

#### 3.2.3 Forces shaping the implementation of GCS

Although policy and governance frameworks were described as essential for legitimizing GCS, participants' accounts indicate that their presence alone was insufficient to guarantee implementation in everyday clinical practice. Organizational pressures, high workloads, and managerial resistance frequently constrained the allocation of time and resources for supervision. In several contexts, GCS was perceived as competing with direct patient care, rather than as an integral component of service quality and professional sustainability.

##### Constraint pressures: Resources, workload, and resistance

Participants described time constraints and staffing shortages as the most immediate barriers to the implementation of GCS in the genetic services. In addition, it was also reported that institutional resistance, with some employers reluctant to allocate time or resources for GCS, perceiving it as secondary to direct patient care. These constraints were said to affect the access and frequency of GCS. For example, a participant from Brazil noted that while training programs often include time for GCS, this is rarely the case in routine clinical practice where management will prefer that all the time is allocated to appointments.

I am a medical geneticist contracted for 20 hours. I won't have a reserved space in my contract to have this kind of support, you know? The city will want me to work 20 hours seeing patients, right? Full time, at least during those 20 hours. I won't have, in my schedule, a reserved slot of 2 or 3 hours every week to participate in the group. Now, if you are a medical resident, or a resident in a multiprofessional genetic counseling program, then you will have a preserved schedule for this. So, I see this happening during the training period, but I don't see it being incorporated into regular practice. (Brazil, MG)

Similarly, a participant from Canada highlighted that management often views supervision as competing with patient care, rather than as an integral component of quality service:

There is also a resistance from management; they see supervision as time away from patients rather than as part of quality care. (Canada, GC)

Participants also highlighted financial arrangements as a key factor shaping access to GCS. In contexts where GCS was institutionally funded, broad accessibility was ensured. Participants also described adaptations to mitigate these barriers such as, for instance, a collegial GCS model that was adopted, allowing colleagues to supervise each other at minimal cost via a digital platform and a simple, structured protocol.

We just recently arranged GCS, but not everyone has the opportunity. So, we recently introduced a collegial GCS, where colleagues supervise each other. The reason we chose this is because it was feasible at a very low cost, as money is always a barrier. It's also accessible to many of our members and even non‐members because we do it digitally, online, and we follow a very simple protocol that a researcher here in Sweden developed for collegial supervision. It is very simple, straightforward, effective, and much appreciated. (Sweden, GC)

Because of convenience, my schedule and the cost involved, I decided to pay for my own supervision with a person from the UK who specialized in this. I also acknowledge that I liked how GCS worked in the UK. (Spain, GC)

Participants described that in the absence of formal GCS, peers or other health and social professionals often provided guidance and support. They noted that while these arrangements were helpful for immediate reflection or case discussion, they were inconsistent, temporary, and dependent on the availability and willingness of colleagues, resulting in variability in the quality and regularity of supervision.

I know what you mean, because I was working in the UK for 2 years and I had GCS there. In Israel we don't have GCS, unfortunately. I think that's really a pity. But we do have some kind of supervision. For example, in my hospital we have a social worker who is part of our team mainly for clients, if a couple needs to decide about termination of pregnancy, for example, and they find it difficult to decide. We sometimes involve her helping them to reach a decision. So, she helps us in a way as well. If we have questions about how best to do things, or you know any other challenges. We will consult with her, but it's not regulated. It is not permanent. (Israel, GC)

So no, there hasn't been GCS, but we are so slowly establishing a route to be able to do this. And we actually have a psychologist at the department that has been quite willing to help us, you know, when we have to talk about something. (Iceland, GC)

##### Enabling forces: Networks, education, and digital solutions

Participants described professional networks and collaborations as vital for supporting and sustaining GCS. They highlighted that those networks provided opportunities for sharing experiences, exchanging knowledge, and learning from peers, as well as avenues for advocacy and political influence to promote recognition and institutional support.

For example, a participant from Austria highlighted the role of international and European networks in shaping professional standards and promoting recognition at a national level:

I think being able to refer to a higher entity, like the European Society of Human Genetics, and to say, see, on the European level these three columns already exist: the medical genetics, the genetic counseling, and the clinical laboratory analysis counselors. These are the three columns of European Society, and then the national societies are like… well, maybe we need to consider as well, right? So, it's really great to have this interconnection. And I think this is what really brings the profession forward, you know, to have a network of people. And I really like that the boards and now also having a joint publication (…). (Austria, GC)

Similarly, a participant from Cyprus described how collaboration with international training programs create opportunities for professional development across different contexts, linking local practice with broader educational initiatives:

We definitely have (GCS) it within our own team, for new genetic counselors, we're there to provide support and we also have been involved in the local training of genetic counselors who were completing the long‐distance MSc programme set up by Cardiff University, which has been a great experience of joining different realities. (Cyprus, GC)

Participants reported introducing GCS into formal education and using digital platforms as strategies to expand access. Integrating GCS into training programs was viewed not only as a way to develop counseling skills but also to cultivate future supervisors, integrating reflective practice as a core element of professional development:

Introducing supervision as part of our core training has been essential. It prepares us not just to be good counselors, but also to be effective supervisors. (Spain, GC)

Participants also highlighted that those digital platforms increased accessibility and flexibility, allowing engagement across diverse locations.

And this is the reason why we chose this GCS online – because it was feasible for a very small cost. It's also accessible to very many of our members and even nonmembers because we do it digitally online and we follow a very a simple protocol that has been a researcher here in Sweden has developed for collegial supervision and she's a nurse practitioner. So, she did it in the beginning for nurses, and I took it, and we've adapted it to us. (Sweden, GC)

##### Genomics and increasing biomedical complexity

Participants reflected on how the rapid expansion of genomic knowledge has significantly increased the complexity of their professional practice. They constructed that the advancements brought “gray area” information, where the clinical significance of many variants remains uncertain, and the increasing volume of data and interpretations presents ongoing challenges. These advances intensify the technical demands of practice and amplify the uncertainty, making the role of GCS increasingly critical in supporting practitioners to navigate complex clinical responsibilities, ethical dilemmas, communication challenges, and evolving professional expectations.

GCS is important because the genetic counseling profession is inherently complex. Every patient, every family brings something emotionally or ethically challenging. No one comes to see us with something light; there's always a serious or difficult story behind it. That's why GCS is not just useful, it's necessary. It gives us space to process and reflect on these situations, to deal with the increasing complexity of genomic information, and to make sure we're supporting patients, and ourselves, in the best way possible. (Australasia, GC)

I think that after the classification of the variants comes the interpretation, but also, how do you communicate it? We already know about this, we already have the knowledge, but I believe we need to go deeper into this field of communication: not only with the patient, but also with doctors and with the laboratory. And then I think that maybe each group could have a genetic counselor to help manage with the ethical dilemmas of the increasing complexity of genomics information. (Spain, GC)

## 4 DISCUSSION

This study aimed to investigate how GCS has been implemented globally, identifying best practices, gaps, and strategies for effective implementation. Overall, our results demonstrate that GCS remains heterogeneously provided and poorly standardized, with no commonly adopted models across the countries analyzed, reflecting fragmented practices worldwide and how different theoretical approaches influence the perception of supervision (Goodyear et al., 1984; Guimarães et al., 2024; Paneque et al., 2023). Yet, despite this variability, there was clear unanimity among respondents that GCS is essential not only for maintaining quality of care but also for supporting counselor well‐being in a context where the risk of burnout is particularly high, both among practicing genetic counselors (Bernhardt et al., 2009) and students undergoing genetic counseling training (McCormick et al., 2025).

Although there is broad recognition of the theoretical value of GCS, its practical implementation remains inconsistent across contexts. While established models, such as the Reciprocal Engagement Model (REM) (Suguitan et al., 2018) and narrative approaches (Ferrer‐Duch, 2025), offer valuable conceptual insights into relational and reflective dimensions of counseling, their direct application to supervision has not yet been systematically theorized or empirically evaluated. Rather than representing established supervision frameworks, these models point to potential directions for future research exploring how counseling theories might be meaningfully adapted to supervisory contexts.

Historical and structural factors, particularly the official recognition of the profession, seem to shape awareness, access, and the institutionalization of GCS. In contexts where the genetic counseling profession lacks formal recognition, structured supervision systems are often limited, as professional efforts focus primarily on securing legitimacy rather than consolidating GCS infrastructures (Guimarães et al., 2024; Paneque et al., 2023). Conversely, countries with an established genetic counseling profession and accreditation mechanisms tend to have more consistent and integrated supervision practices (Canton et al., 2024; Paneque et al., 2023). Regulatory frameworks, including defined standards of practice and formal registration requirements, decisively seem to shape professional trajectories and facilitate the integration of GCS into institutional policies (Guimarães et al., 2024; McEwen et al., 2025).

A clear illustration of this dynamic comes from the United States, whereby in the late 1990s and early 2000s, the genetic counseling profession had achieved significant institutionalization through accredited training programs, professional associations, and credentialing mechanisms. Within this context, Kennedy (2000) described structured supervision models for practicing genetic counselors, including leader‐led supervision groups, demonstrating how professional maturity enables the development of systematic GCS frameworks. Similarly, Weil (2000) emphasized the central role of supervision in supporting practicing counselors, highlighting the need for structured and ongoing guidance to ensure professional development and high‐quality care. These examples, dating back 25 years, illustrate how the evolution of the profession and the establishment of supervision systems are intricately linked and evolve as a unified process.

The patterns observed in the USA are mirrored internationally: robust professional status facilitates inclusion in healthcare policies, enabling sustainable funding and resource allocation for GCS. Governance frameworks and professional networks, such as the ESHG, NSGC, HGSA and GCRB, further support supervision by promoting standardization, providing resources, and offering guidance for shared learning and professional support (European Society of Human Genetics, 2018; Genetic Counseling Registration Board, 2022; Human Genetics Society of Australasia, 2018; National Society of Genetic Counselors, n.d.).

On the other hand, competence‐based educational pathways strengthen the link between professional maturity and supervision, as introducing GCS within academic and clinical training fosters early recognition of its importance and cultivates professionals who view supervision as integral to ethical and reflective practice (Costa et al., 2025; Gonsalvez & Calvert, 2020). Reflective, long‐term supervision supports continuous professional development, contributes to counselor resilience, and enhances the overall growth and robustness of the profession internationally (McEwen et al., 2025).

Despite these advances, evidence on how supervision frameworks operate across diverse cultural and institutional contexts remains limited, highlighting the need for systematic documentation and comparative evaluation of national strategies. Across contexts, there is strong consensus that GCS is central to improving practice, supporting practitioner well‐being, preventing burnout, and enhancing the quality of genomic healthcare (Guimarães et al., 2024; McEwen et al., 2025; Paneque et al., 2023). Institutionalizing supervision as a standard component of professional practice ensures both individual and collective benefits by reinforcing counselor resilience, fostering continuous learning, and strengthening the profession globally. However, challenges persist, including financial and human resource constraints, shortages of experienced supervisors, heavy clinical workloads, and institutional resistance (Berg et al., 2018; Costa et al., 2024; Cox, 2022; McEwen et al., 2025; Rothwell et al., 2021). These challenges require strategic policy engagement, dedicated resources, and systematic documentation of both successful and less successful implementation pathways.

Creating a global network could be an approach to support the development of GCS, particularly in countries where supervision resources are scarce. By connecting supervisors and supervisees across borders, such a network could foster knowledge mentorship and collaborative learning on an unprecedented scale. However, implementing this model would also introduce significant challenges (Gu et al., 2011). Cultural differences, language, and variations in professional standards would introduce an additional layer of complexity that requires careful adaptation and contextual sensitivity. Developing a feasible and equitable global network would therefore demand strategic planning and ongoing evaluation to ensure that it enhances GCS access without exacerbating existing inequalities.

### 4.1 Limitations

Our purposive sample of senior representatives provided insight into policy and implementation issues but excludes the experiences of trainees and early‐career counselors. Future research should include these perspectives. Interviews were mainly in English and transcripts were analyzed in original languages and translated excerpts were double‐checked by the research team, but language differences remain a limitation. Coverage was broad but uneven across continents (stronger representation from Europe). Some countries were reported regionally. Finally, as a reflexive thematic study, findings reflect the interpretive stances of the research team; we have therefore embedded positionality and reflexive notes throughout Methods and provided a RTA checklist in the Supplement.

## 5 CONCLUSION

In conclusion, although GCS practices remain heterogeneous across countries and healthcare systems, there is broad agreement regarding their perceived value and a growing recognition of the need for stronger empirical evidence on their outcomes. The findings of this study suggest that effective GCS cannot be understood in isolation from the broader evolution of the genetic counseling profession. They also highlight the urgent need to integrate GCS more systematically into health policies, training programs, and clinical practice, recognizing it as an essential component of quality care. Future efforts should in fact prioritize the development of global collaborative models, including international networks and digital GCS platforms, to reduce inequities in access, particularly in resource‐limited settings. In parallel, structured training, as well as systematic evaluation of GCS's impact on care quality and professional well‐being, will be key. Finally, culturally sensitive and flexible implementation strategies can help promote equitable and consistent integration of GCS worldwide, consolidating its role as a central piece in genetic counseling practice. By investing in structured GCS, health systems can strengthen workforce resilience and enhance genetics quality of care worldwide.

## AUTHOR CONTRIBUTIONS

**Lídia Guimarães:** Conceptualization; formal analysis; investigation; methodology; writing – original draft; writing – review and editing. **Bibiana Ribeiro:** Study planning and design, research, data analysis, and final manuscript revision. **Margarida Rangel Henriques:** Conceptualization; methodology; writing – review. **Marina Serra Lemos:** Conceptualization; methodology, writing – review. **Milena Paneque:** Conceptualization; formal analysis; investigation; methodology; writing – review and editing; supervision.

## FUNDING INFORMATION

This study was developed with the support of a PhD Scholarship from the Foundation for Science and Technology (Fundação para a Ciência e Tecnologia) with the reference FCT‐2023.00348.BD, attributed to Lídia Guimarães.

## CONFLICT OF INTEREST STATEMENT

All authors declare that they have no conflicts of interest.

## ETHICS STATEMENT

Ethical approval for this study was obtained from i3S – Institute for Research and Innovation in Health.

Human studies and informed consent: Informed consent was obtained from all the participants in this study.

Animal studies: We did not refer to animal studies, as this does not apply.

## POSITIONALITY

As researchers, we acknowledge that our social, professional, and cultural positions may influence both the data collection process and the interpretation of results. Our team is composed of women with diverse academic backgrounds and expertise, including psychology, biology, and genetic counseling, with members spanning distinct levels, from students to professors, bringing a wide range of perspectives and professional experiences to the study. Our distinct cultural contexts, shaped by our backgrounds in Portugal and Cuba, have also influenced our understanding of genetic counseling. Reflecting on our positionality is an ongoing effort to ensure that our interpretations are as unbiased as possible, while acknowledging that our perspectives, like any others, play a role in the construction of knowledge.

## Supporting information


Appendix S1

## Data Availability

The data that support the findings of this study are available on request from the corresponding author. The data are not publicly available due to privacy or ethical restrictions.
