# Supplementary material for: The state of genetic counseling supervision worldwide: Challenges, practices, and pathways for the future
Source: J Genet Couns. 2026 Jan 21;35(1):e70169. doi: 10.1002/jgc4.70169 (PMC12821561; doi:10.1002/jgc4.70169)
Supplement: Supplementary file 1 — Appendix S1 [file JGC4-35-0-s001.doc]

**Appendix 1**

**Sociodemographic Data Questionnaire**

1. Country/countries you will be representing
2. Main professional activity:
   1. Genetic counsellor
   2. Medical geneticista
   3. Other:
3. Years of experience in the field of Genetics
   1. 0-10
   2. 11-20
   3. +21
4. Which of the following most accurately describes your profile regarding the eligibility for this research study:
   1. Representative member of a genetic counsellors professional Association
   2. Representative member of an international Board
   3. Founder of a genetic counselling master's programs
   4. Advocate for the profession's recognition in the country/ies
   5. Senior genetic counsellor
   6. Senior geneticista
   7. Other
